# Presynaptic Proteins as Markers of the Neurotoxic Activity of BmjeTX-I and BmjeTX-II Toxins from* Bothrops marajoensis* (Marajó Lancehead) Snake Venom

**DOI:** 10.1155/2016/2053459

**Published:** 2016-08-18

**Authors:** Antonio Lisboa, Rodolfo Melaré, Junia R. B. Franco, Carolina V. Bis, Marta Gracia, Luis A. Ponce-Soto, Sérgio Marangoni, Léa Rodrigues-Simioni, Maria Alice da Cruz-Höfling, Thalita Rocha

**Affiliations:** ^1^Multidisciplinary Research Laboratory, São Francisco University (USF), Avenida São Francisco de Assis 218, Jardim São José, 12916-350 Bragança Paulista, SP, Brazil; ^2^Department of Biochemistry and Tissue Biology, Institute of Biology, State University of Campinas (UNICAMP), Rua Monteiro Lobato, 255, Cidade Universitária Zeferino Vaz, 13083-365 Campinas, SP, Brazil; ^3^Department of Pharmacology, Faculty of Medical Sciences, State University of Campinas (UNICAMP), Rua Tessália Vieira de Camargo 126, Cidade Universitária Zeferino Vaz, 13083-881 Campinas, SP, Brazil

## Abstract

Neuromuscular preparations exposed to* B. marajoensis* venom show increases in the frequency of miniature end-plate potentials and twitch tension facilitation followed by presynaptic neuromuscular paralysis, without evidences of muscle damage. Considering that presynaptic toxins interfere into the machinery involved in neurotransmitter release (synaptophysin, synaptobrevin, and SNAP25 proteins), the main objective of this communication is to analyze, by immunofluorescence and western blotting, the expression of the synaptic proteins, synaptophysin, synaptobrevin, and SNAP25 and by myography, light, and transmission electron microscopy the pathology of motor nerve terminals and skeletal muscle fibres of chick biventer cervicis preparations (CBC) exposed* in vitro* to BmjeTX-I and BmjeTX-II toxins from* B. marajoensis* venom. CBC incubated with toxins showed irreversible twitch tension blockade and unaffected KCl- and ACh-evoked contractures, and the positive colabelling of acetylcholine receptors confirmed that their action was primarily at the motor nerve terminal. Hypercontraction and loose myofilaments and synaptic vesicle depletion and motor nerve damage indicated that the toxins displayed both myotoxic and neurotoxic effect. The blockade resulted from interference on synaptophysin, synaptobrevin, and SNAP25 proteins leading to the conclusion that BmjeTX-I and BmjeTX-II affected neurotransmitter release machinery by preventing the docking of synaptic vesicles to the axolemma of the nerve terminal.

## 1. Introduction

In Brazil, the accidents caused by* Bothrops* snakes are close to 74% according to the National System of Accidents Notification (SINAN) [1]; they constitute a major public health problem in Brazil. Great part of these accidents occurs at remote country areas far from appropriate first aid intervention [2]. Generally, the envenomation picture is characterized by pronounced myonecrosis [3–5] and systemic effects [6]. Such effects result from proteolytic, myotoxic, blood-clotting, and hemorrhagic activities attributed to the phospholipases A_2_ contained in their venoms. One dominant effect of* Bothrops* snakebites is marked myotoxicity without any clinical sign of neurotoxicity. However,* in vitro* studies in amphibian, avian, and mammalian nerve-muscle preparations have shown neurotoxicity, with total and/or irreversible neuromuscular blockade, at very low concentrations of venom [7, 8] or toxins [8–10] from different species. Hence, it is likely that a direct action of phospholipase A_2_ (PLA_2_) myotoxins plus an indirect action caused by tissue anoxia has key roles in the effects of* Bothrops* venom on the muscle and nerve fibres, including immediate destruction of motor axons and complete depletion of intramuscular motor nerve trunks [11].

**Figure 1:**
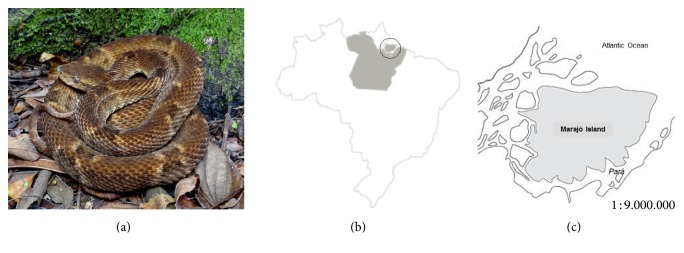
(a) Image of an adult male specimen of* Bothrops marajoensis* (photo: Silvia Cardoso, Ph.D.; Museu Biológico/Instituto Butantan, São Paulo, SP, Brazil). (b) The Brazilian map with Pará state in gray and (c) an enlarged detail of the Marajó Island and costal drainage at the Amazon estuary.

Brazilian species of the* Bothrops* genus are close to 50. Geographically, same species can be distributed all over the country while others are endemic, for example,* Bothrops marajoensis,* which is only found in the Marajó Island, Pará State, in the very North of Brazil [12, 13] (Figure 1).

Notified accidents with* B. marajoensis* are rare; a first case of hemorrhagic stroke in a child bitten by* B. marajoensis* in Anajás city, Marajó Island, was recently reported; the permanent hemiplegia sequela suffered by the victim was attributed to delayed medical intervention [14]. Also, an accident occurred with a specimen in captivity that bit the right hand thumb of the snakes' caretaker in the Instituto Butantan; the bite produced immediate excruciating pain and edema, which spread rapidly to her hand and part of the forearm leading to ecchymosis and blister formation. The intense pain and throbs persisted for 48 hours despite rapid medical care at the Hospital Vital Brazil (Silvia Cardoso, Ph.D., personal communication, Instituto Butantan).

In neuromuscular preparations of chick biventer cervicis (CBC) and mouse phrenic nerve-diaphragm (PND), concentrations as low as 1 or 5 *μ*g/mL of* B. marajoensis* crude venom elicited increases in the frequency of miniature end-plate potentials (MEPPs) which occurs concomitantly with twitch tension facilitation followed by presynaptic neuromuscular paralysis but without evidences of muscle damage [15].

Three basic Asp-49 phospholipases A_2_ (PLA_2_) have been isolated from* B. marajoensis* venom: Bmaj-9 (13679.33 Da) [16], BmjeTX-I (13808.89 Da), and BmjeTX-II (13863.97 Da) PLA_2_s [17] and were shown to act presynaptically in CBC preparations. At appropriate concentrations, BmjeTX-I and BmjeTX-II induce neuromuscular junction blockade at presynaptic sites with no response elicited in proper muscle and also myonecrosis with systemic interleukin-6 response, moderate marked paw, and cytotoxicity in murine skeletal muscle C2C12 myoblasts and myotubes [17].

Venom's toxins that act on presynaptic sites interfere in complex machinery where proteins, such as synaptophysin, synaptobrevin, and SNAP25, undertake specific roles at precise steps of neurotransmitter release. Synaptophysin, an integral protein of the synaptic vesicle, binds to synaptobrevin and may act to prevent vesicle docking at the axolemma of the terminal bouton; SNAP25 is a plasma membrane protein (t-SNARE), which in association with synaptobrevin forms a stable complex responsible for vesicle docking, priming, and fusion [17]. Alterations in this complex can affect the neurotransmitter release and induce a presynaptic-blocking effect.

In this communication we describe our observations on the cellular pathology of the motor nerve terminal and skeletal muscle fibres exposed* in vitro* to two major toxins isolated from the venom of* B. marajoensis*. Our hypothesis is that BmjeTX-I and BmjeTX-II affect neurotransmitter release machinery by preventing the docking of synaptic vesicles to the axolemma of the nerve terminal.

## 2. Material and Methods

### 2.1. Animals

Male HY-line W36 chicks (4–8-day-old) were supplied by Globo Aves Agrovicultura Ltda (Campinas, SP, Brazil). Animals were housed at 25°C under a 12 h light/dark cycle with free access to food and water. All procedures were approved by the Institutional Committee for Ethics in Animal Use (CEUA/São Francisco University, protocol number 000.11.10) and are in accordance with the Brazilian Society of Laboratory Animal Science (SBCAL/COBEA) guidelines.

### 2.2. Toxins and Reagents

BmjeTX-I and BmjeTX-II isolated from* B*.* marajoensis* snake venom were purified as described in full detail by Ponce-Soto et al. [17].

All reagents were obtained from Sigma-Aldrich (MO, USA) if not stated otherwise. Rabbit anti-glyceraldehyde-3-phosphate dehydrogenase (GAPDH-FL335) was from Santa Cruz Biotechnology (Santa Cruz, CA, USA); OCT-Tissue Tek was from Sakura Finetek. EPON EMBed-812 Kit, uranyl acetate, osmium tetroxide, lead citrate, and glutaraldehyde were from Electron Microscopy Sciences (Hatfield, PA, USA). Western blotting reagents were purchased from Sigma-Aldrich, Biorad, Amresco, and Kodak. All salts for the physiological solution were of analytical or sequencing grade.

### 2.3. Chick Biventer Cervicis (CBC) Preparation

Chicks (*n* = 12) were killed by halothane inhalation and immediately the biventer cervicis muscles were removed and mounted under a tension of 1 g in a 5 mL organ bath containing Krebs solution (composition in mM: NaCl 118.7, KCl 4.7, CaCl_2_ 1.88, KH_2_PO_4_ 1.17, MgSO_4_ 1.17, NaHCO_3_ 25, and glucose 11.65), pH 7.5 at 37°C, and carbogen aeration (95%/5% O_2_/CO_2_, v/v) [18] for twitch tension recordings. The preparations were allowed to stabilize for at least 20 min before addition of a single concentration of BmjeTX-I or BmjeTX-II PLA_2_ toxins (10 *μ*g/mL). Such concentration was the same used by Ponce-Soto et al. [17].

A bipolar platinum ring electrode was placed around the muscle and coupled to a Grass S48 stimulator (0.1 Hz, 0.2 ms duration, 4–8 V). Isometric muscle contractions and contractures were recorded via a force displacement transducer (Load Cell BG-50 Grams, Kulite Semiconductor Products, Inc.) coupled to a physiograph (Gould, Model RS 3400). Contractures to exogenous acetylcholine (ACh, 110 *μ*M) and potassium chloride (KCl, 40 mM) were obtained in the absence of field stimulation prior to toxins addition and by the end of the experiment (120 min).

### 2.4. Morphological Analysis by Light Microscopy

Longitudinal cryosections of CBC (*n* = 4), maintained in carbogen-aerated Krebs solution (control) or incubated with 10 *μ*g/mL of BmjeTX-I or BmjeTX-II for 120 minutes under indirect stimulation, were collected onto subbed glass slides, permeabilized in ethanol and methanol (−20°C, 10 min each bath), rinsed with distilled water, and stained with haematoxylin and eosin (HE) for histological analyses.

### 2.5. Morphological Analysis by Transmission Electron Microscopy (TEM)

Samples containing end-plate regions of CBC (*n* = 4), maintained in aerated Krebs solution (control) or incubated with 10 *μ*g/mL of BmjeTX-I or BmjeTX-II for 120 minutes under indirect stimulation, were pinned side by side to wax under slight longitudinal tension, immersion-fixed in Karnovsky's solution for 30 minutes at room temperature and overnight at 4°C. Afterwards, the samples were washed in 0.1 M sodium cacodylate buffer, post-fixed in 1% OsO_4_ solution for 2 h, washed in the same buffer followed by distilled water, contrasted with 5% uranyl acetate for 1 h, washed in distilled water, dehydrated in acetone series, and embedded in Epon resin (Epon : acetone 2 : 1, 1 : 1, and 1 : 2 and pure EPON). Ultrathin sections (60 nm thick) stained with uranyl acetate followed by lead citrate were examined in a Leo 906 transmission electron microscope (Zeiss, Oberkochen, Germany). The ultrastructure of the muscle fibres and neuromuscular junctions and morphometry of synaptic vesicles were provided.

### 2.6. Immunofluorescence (IF) Analysis

Transversal cryosections of CBC (*n* = 4 animals), maintained in carbogen-aerated Krebs solution (control) or incubated with 10 *μ*g/mL of BmjeTX-I or BmjeTX-II for 120 minutes under indirect stimulation, were collected onto subbed glass slides, permeabilized in ethanol and methanol (−20°C, 10 min each), and in 0.1% Triton X-100 in phosphate buffered saline (PBS) (15 min, room temperature), rinsed with PBS, and incubated overnight in a moist chamber at 4°C with appropriate primary antibodies (diluted to a final concentration of 1/150 synaptophysin, 1/250 SNAP25, and 1/300 synaptobrevin). Control of the immunoreaction was done by omitting the primary antibodies. On the following day, the slides were allowed to return to room temperature, washed in PBS, and incubated with the appropriate FITC-conjugated secondary antibody (goat anti-rabbit 1/80, rabbit anti-goat 1/800, and goat anti-mouse 1/100) or TRITC-conjugated *α*-BgTX (diluted to a half secondary antibody concentration) for 2 h at room temperature and then mounted in glycerin jelly. The positive immunolabelling was accessed, using a fluorescence BX51TF microscope (Olympus Optical Co. Ltd., Tokyo, Japan), from 6 serial sections and the positive reactions were counted for statistical purposes (morphometry).

### 2.7. Western Blotting (WB)

CBC nerve-muscle preparation (*n* = 4), maintained in carbogen-aerated Krebs solution (control) or incubated with 10 *μ*g/mL of BmjeTX-I or BmjeTX-II for 120 minutes under indirect stimulation, were homogenized in 1 mL of antiprotease cocktail. Aliquots of 15 *μ*g proteins were used for 12% sodium dodecyl sulphate polyacrylamide gel electrophoresis (120 V, 90 min). After electrophoretic proteins transfer to nitrocellulose membrane (400 mA, 90 min), the samples were blocked overnight at 4°C in PBS containing 5% w/v dried milk and probed with primary antibodies (diluted in PBS containing 3% w/v dried milk to a final concentration of 1/500 synaptophysin, 1/500 synaptobrevin, and 1/1000 SNAP25) for 4 h. Blots were washed in PBS and incubated with a corresponding HRP-conjugated secondary antibody diluted in PBS containing 1% w/v dried milk to a final concentration of 1/1000 goat anti-rabbit, 1 : 5000 rabbit anti-mouse, and 1 : 3000 rabbit anti-goat, respectively.

The blots were scanned, stored as TIFF files, and quantified using Image J 1.45s software (Wayne Rasband, NIH, Bethesda, MD, USA). Densitometric data of endogenous control were generated by incubating blots with GAPDH (1/1000, followed by 1/1000 goat HRP-conjugated anti-rabbit). After rinsing in PBS, the immunoreactive bands were detected by chemiluminescence (Super Signal, Pierce West Pico Chemiluminescent Substrate, USA) using X-ray film (BioMax XAR Film Kodak, USA). Experimental data were expressed in terms of relative optical density.

### 2.8. Statistics

Quantitative data were expressed as mean ± standard deviation (SD). Statistical significance was determined by one-way ANOVA followed by the Bonferroni* post hoc* test with *p* < 0.05 indicating significance. All analyses were done using Prism software (GraphPad Inc., San Diego, CA, USA).

## 3. Results

### 3.1. Myography

**Figure 2:**
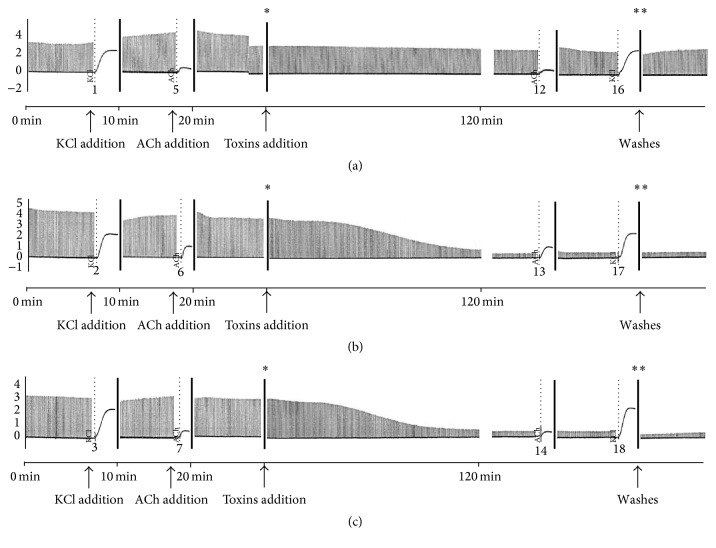
Chick biventer cervicis preparations in Krebs (control, (a)), or incubated with 10 *μ*g/mL of BmjeTX-I (b) and with 10 *μ*g/mL of BmjeTX-II (c) toxins isolated from* B. marajoensis* crude venom. Notice, in (b) and (c), the irreversible neuromuscular blockade induced after 31.2 ± 3.5 min and 30 ± 8.1 min (*p* < 0.05 from Krebs) of toxins (**∗**) addition, respectively. ACh and KCl addition; *∗∗*: washes (*n* = 12 for each treatment).

CBC preparations incubated with BmjeTX-I or BmjeTX-II (10 *μ*g/mL) displayed total and irreversible neuromuscular blockade after 31.2 ± 3.5 min and 30 ± 8.1 min (*p* < 0.05 from Krebs), respectively, similarly to previous report [16]. The neuromuscular effects of both PLA_2_ toxin isoforms resulted from increase in the twitch response of indirectly stimulated neuromuscular preparations. None of the toxins interfered significantly with the contractures to exogenously applied ACh and KCl after 120 min incubation (*n* = 12) (Figure 2).

### 3.2. Muscle Morphology and Ultrastructural Analysis

**Figure 3:**
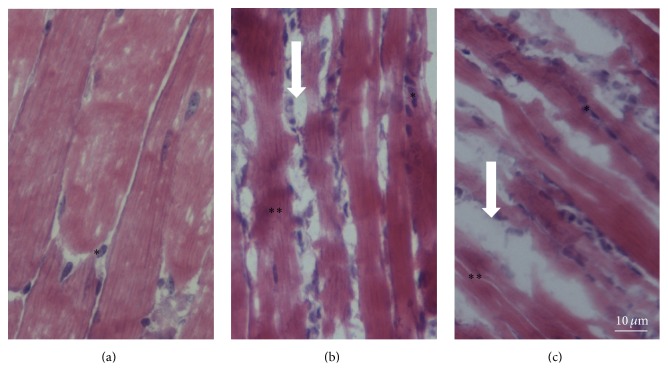
Light micrographs of chick biventer cervicis preparations incubated with Krebs solution (control, (a)) or incubated with BmjeTX-I (b) and BjmeTX-II (c) (10 *μ*g/mL each). All images were obtained from longitudinal sections. Notice that in (a) the fibres are normal in appearance, while in (b) and (c) several fibres appear with disrupted myofibrils (white arrows) and hypercontracted zones throughout their length (*∗∗*) and nucleus (*∗*); HE, *n* = 4 per treatment.

Light microscopy showed that control CBC (incubated for 120 min in Krebs solution) presented muscle fibres with normal morphology while CBC incubated with BmjeTX-I or BmjeTX-II presented regions with fibres with different pathologic states, including vacuolated or swollen fibres, presenting loosely and/or densely clumping of myofibrils (Figure 3) but also regions with normality (not shown).

**Figure 4:**
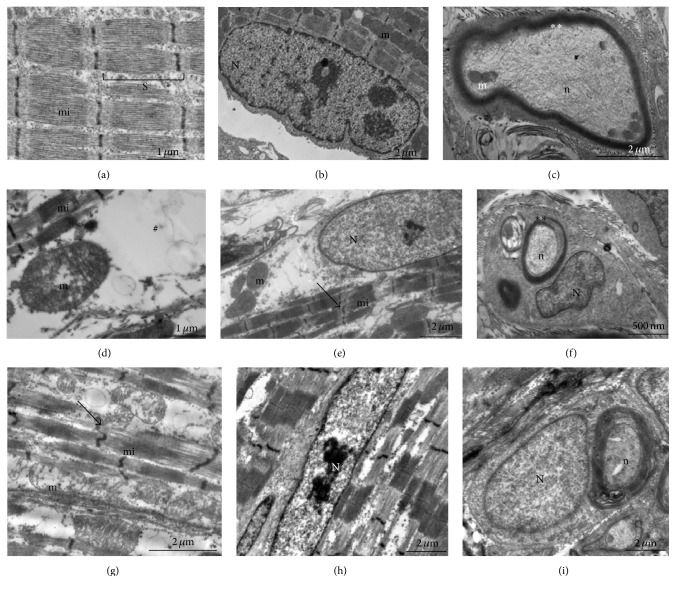
Electron micrographs of chick biventer cervicis preparations incubated in Krebs solution (control, (a)–(c)), BmjeTX-I ((d)–(f)), and BmjeTX-II ((g)–(i)) toxins. No alterations were observed in control ((a)–(c)). Treated preparations ((d)–(i)) displayed hypercontracted myofilaments (mi) and loss of typical sarcomeres (S) organization, edema (#), mitochondria (m), swelling, and cristae deprivation. Myonuclei (N) of some fibres showed apoptotic-like phenotype and intramuscular nerve fibres were affected (*∗∗*); n: neurofilaments; arrows: blurred Z line (*n* = 4 per treatment).

Transmission electron microscopy was used to assess whether BmjeTX-I and BmjeTX-II affect the subcellular integrity of muscle fibres and neuromuscular junction (NMJ) of biventer cervicis. Control muscles showed typical ultrastructure with paralleled myofibrils and organized sarcomeres. Longitudinal- and cross-sectioned myofibrils were typically separated by profiles of sarcoplasmic reticulum; subsarcolemmal nuclei and mitochondria were observed without any damage. Intramuscular axons showed well organized myelin sheath and normal neurofilaments and mitochondria (Figures 4(a)–4(c)).

Opposed to controls, CBC preparations incubated either with BmjeTX-I or BmjeTX-II presented hypercontracted fibres and clusters of swollen mitochondria and/or deprived of cristae, blurred Z line with loss of myofilaments, and sarcomeres disorganization. Diffuse swelling of the sarcotubular system was also observed and some fibres exhibited myonuclei with chromatin and nucleoli alterations. Intramuscular motor nerve fibres displayed multishaped alterations of the myelin sheath and axons (Figures 4(d)–4(i)). Regions with normal ultrastructure were also present (not shown).

**Figure 5:**
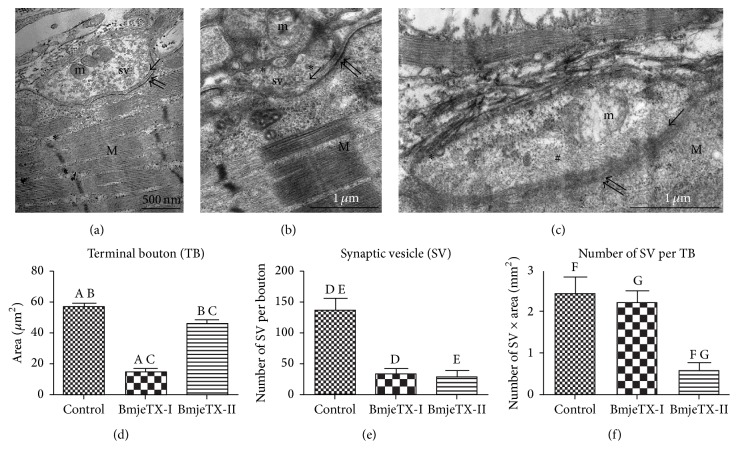
Electron micrographs of neuromuscular junction (NMJ) and synaptic morphometry of control (a), BmjeTX-I-treated (b), and BmjeTX-II-treated (c) chick biventer cervicis preparations. (a) Observe the bouton filled with synaptic vesicles (sv), intact mitochondria (m), and presynaptic membrane (arrow) associated with an unfolded but highly contrasted postsynaptic membrane due to the presence of acetylcholine receptors (double arrow). (b, c) Distorted terminal button, undefined axolemma (arrow), reduction of synaptic vesicles (#), and swollen mitochondria are present in the terminals; M: muscle fibre. (d)–(f) Graphical demonstration of terminal bouton area (d), mean number of synaptic vesicles per bouton (e), and relative number of synaptic vesicles per terminal bouton area (f) for control, BmjeTX-I, and BmjeTX-II groups. The bars with same uppercase letters indicate that there was significant difference (^A, C, D, E^ *p* < 0.0001; ^B, F^ *p* < 0.001; ^G^ *p* < 0.05); data are mean ± SD one-way ANOVA plus Bonferroni posttest (*n* = 4 per treatment).

In order to support the twitch tension findings which showed that BmjeTX-I and BmjeTX-II PLA_2_ caused a presynaptic neuromuscular blockade, we further investigated the neuromuscular junction (NMJ) ultrastructure by TEM. Control samples incubated with Krebs solution showed no abnormal pre- and postsynaptic ultrastructure. The terminal bouton was clearly defined by a continuous axolemma; it was adjusted into a well-delineated synaptic gutter and covered by processes of the Schwann cell. As usual, the synaptic vesicles and mitochondria were polarized to presynaptic axolemma and Schwann cell, respectively; typically, the postsynaptic sarcolemma was unfolded in chick muscle; densities along its length were due to intramembranous presence of postsynaptic receptors (Figure 5(a)).

By contrast, NMJs in the muscles were exposed to BmjeTX-I and BmjeTX-II, despite a number of them exhibiting morphology similar to controls; many others exhibited a clear and significant (*p* < 0.05) reduction in the density of synaptic vesicles inside the terminal bouton while the remaining vesicles were clumped together in small aggregates; a massive mitochondrial damage and absence of presynaptic membrane were also observed (Figures 5(b) and 5(c)). The terminal bouton area and the quantity of synaptic vesicles per area were significantly reduced as compared to control (*p* < 0.05) (Figures 5(d)–5(f)).

### 3.3. Immunofluorescence of Presynaptic Proteins

**Figure 6:**
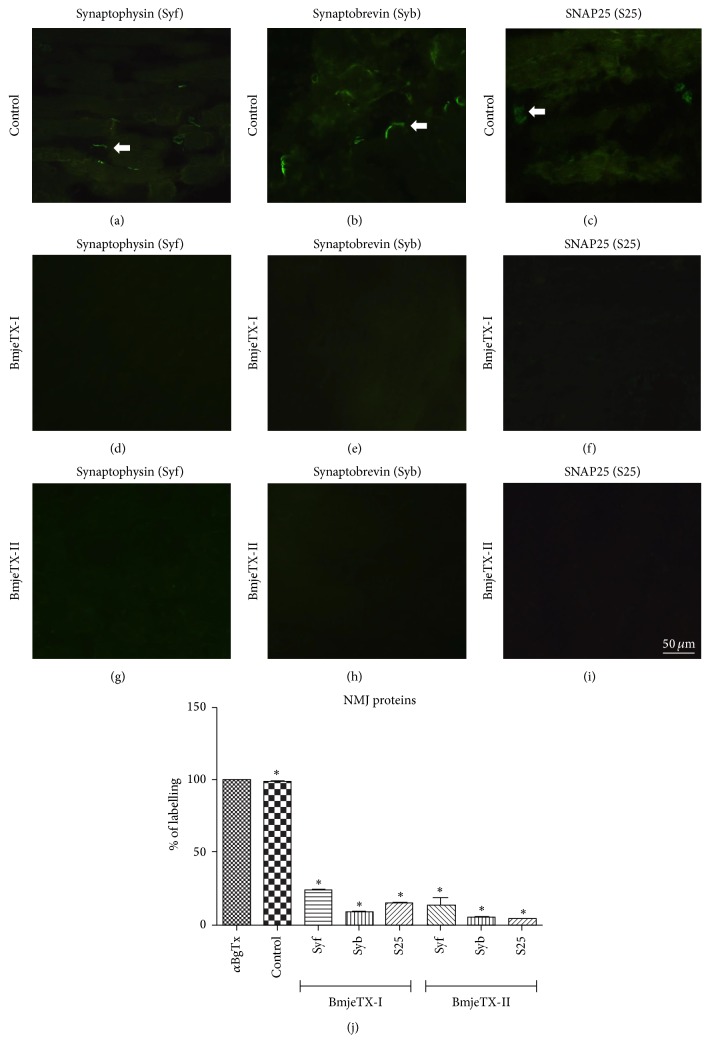
Immunofluorescence for synaptophysin, Syf (a, d, g), synaptobrevin, Syb (b, e, h), and SNAP25, S25 (c, f, i) in neuromuscular junction (NMJ) of chick biventer cervicis. In controls ((a)–(c)) all proteins were expressed indicating active presynaptic machinery. However, in BmjeTX-I-treated ((d)–(f)) and BmjeTX-II-treated (g)–(i) preparations no immunolabelling was detected indicating inactivity of the presynaptic machinery. (j) Percentage of Syf, Syb, and S25 protein showed 100% expression in control preparations and represented as a single control bar, whereas a remarkable reduction was observed for BmjeTX-I- and BmjeTX-II-treated samples. *α*-Bungarotoxin (*α*BgTx) was used to indicate the ACh receptor. Data were expressed as mean ± SD (^*∗*^ *p* < 0.0001 relative to control), one-way ANOVA plus Bonferroni posttest (*n* = 4 per treatment).

Control terminals of biventer cervicis nerve-muscle preparation showed immunolabelling of synaptophysin, synaptobrevin, and SNAP25 proteins (Figures 6(a)–6(c)) while their counterparts incubated with BmjeTX-I or BmjeTX-II showed very weak expression for all three proteins. Figures 6(d)–6(i) illustrate regions of neuromuscular contacts in which labelling is very faint.

The proteins examined showed a 100% basal expression in control, whereas they showed a scale proportion where synaptophysin > SNAP25 > synaptobrevin in preparations incubated with BmjeTX-I and synaptophysin ≥ synaptobrevin = SNAP25 in preparations incubated with BjmeTX-II (Figure 6(j)). The ACh receptors into the postsynaptic membrane, marked with TRITC-conjugated *α*-bungarotoxin (*α*-BgTX), were well preserved for all experimental groups (data not shown).

### 3.4. Western Blotting of Presynaptic Proteins

**Figure 7:**
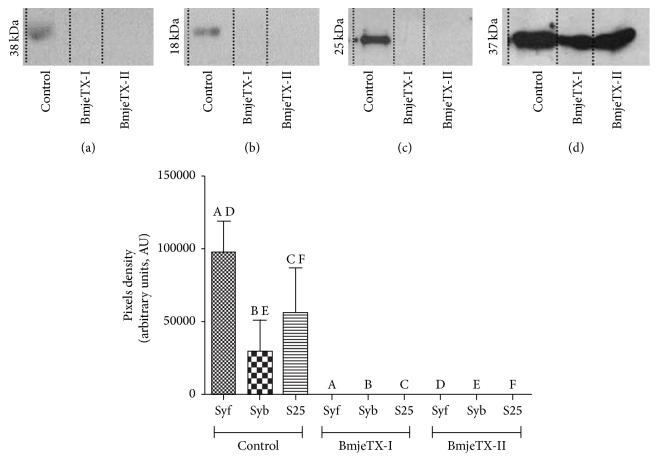
Western blotting analysis for synaptophysin ((a), Syf), synaptobrevin ((b), Syb), and SNAP25 ((c), S25) protein expression in control, BmjeTX-I, and BmjeTX-II chick biventer cervicis preparations normalized to GAPDH (d). The bars with same uppercase letters indicate that there was significant difference: ^A, D^ *p* < 0.001; ^C, F^ *p* < 0.001; ^B, E^ *p* < 0.05. Data were expressed as mean ± SD; one-way ANOVA plus Bonferroni posttest (*n* = 4 per treatment).

Control CBC preparation homogenate showed differential proportion among the three presynaptic proteins examined. The baseline of synaptophysin was double that of SNAP25's which by its turn doubles the baseline of synaptobrevin. In contrast, no expression of synaptophysin, synaptobrevin, or SNAP25 protein was found in homogenate of CBC incubated with BmjeTX-I or BmjeTX-II. Significant differences (*p* < 0.01) were observed between control and PLA_2_s-incubated preparations (Figure 7).

## 4. Discussion

In the present study we have used two PLA_2_s purified from* B*.* marajoensis* venom, BmjeTX-I and BmjeTX-II [17], which as for the venom [15] act presynaptically and possess neuromuscular blocking effect [17]. Our goal was to evaluate the involvement of proteins of the presynaptic apparatus in such effect by comparing their content in CBC control preparations with their content in matched preparations incubated with the two PLA_2_s toxins at a same concentration.

The study was undertaken in* in vitro* preparations, which typically provide faster and reliable information on the effects on neuromuscular junction components. Besides, in muscle-nerve preparation incubation muscle and motor nerve fibres were in closer contact with the toxin through their length instead of a limited tissue portion as when the toxin is injected intramuscularly.

Chick biventer cervicis (CBC) preparation incubated with either of the toxins showed irreversible twitch tension blockade. The KCl- and ACh-evoked contracture was unaffected by either of the PLA_2_s isoforms indicating that at the concentration used the effect in the nicotinic receptors was absent; instead it indicates that BmjeTX-I and BmjeTX-II (10 *μ*g/mL) action was primarily at the motor nerve terminal, as suggested elsewhere [17]. Bmaj-9, another presynaptic-acting PLA_2_ from* B*.* marajoensis* venom, likewise induced a total and irreversible blockade after 70 ± 5 min at same concentration and same nerve-muscle preparation; likewise, the contracture evoked by KCl and ACh remained unchanged by the Bmaj-9 [16].

In agreement, 1 *μ*g/mL low concentration of* B*.* marajoensis* venom was able to induce neuromuscular blockade without depressing the responses to exogenous ACh and KCl, blocking postsynaptic acetylcholine receptors or interfering with the muscle contractile mechanisms [15]. Nevertheless, concentration as high as 20 *μ*g/mL produced total blockade at around 100 min [15] and promoted significant reduction of KCl- and ACh-induced contractures. The data show the importance of concentration to disclose myotoxic and neurotoxic effect of venom.

Based on the data, it can be concluded that the presynaptic action of the venom could be on account of Bmaj-9 [16], BmjeTX-I, and BmjeTX-II [17] and that the presynaptic-blocking effect is higher with BmjeTX-I and BmjeTX-II than with Bmaj-9. Interestingly, the presynaptic blockade is achieved more fastly whenever each of the three toxins is used than with the whole venom. A substantiated explanation for this was not supported by our findings; we can just speculate that other venom components probably antagonize the effects of the PLA_2_ toxins.

The blockade of the twitch tension in CBC preparations incubated with BmjeTX-I or BmjeTX-II was found to occur in connection with the shift of synaptophysin, synaptobrevin, and SNAP25 proteins from the biventer cervicis end-plates relative to the control counterparts which have shown total expression of the three proteins (IHC and WB data). In control CBC incubated with Krebs solution the immunolabelling of presynaptic proteins delineated their distribution in the synaptic contacts over the myofibres indicating an operative biventer cervicis contractile structure which was responsive to the indirect electric nerve stimulation; these data and the colabelling of acetylcholine receptors (AChR) as proved by *α*-bungarotoxin labelling even in treated preparations (figure not shown) confirmed the normal and functional pattern of the postsynaptic machinery in controls and postsynaptic intactness in treated preparations (not shown).

Complex presynaptic machinery is necessary to release the neurotransmitter at the synaptic cleft of the neuromuscular junction. Initially the synaptic vesicles (SV) are attached to a fine, filamentous actin cytoskeletal network in the presynaptic portion of the nerve terminal. The neurotransmitter vesicle binds to an active target zone near calcium channels (tethering) and docks to the presynaptic membrane and an ATPase-dependent process facilitates the SV priming. Once primed the SV can fuse and release the neurotransmitter. This process is mediated by vesicle-associated, cytosolic, and membranous proteins [19].

Another important finding of this study was that the area of the terminals was significantly reduced in preparations incubated with Bjme-TX-I and Bjme-TX-II with reduction significantly higher with the former than with the latter. Moreover, the number of synaptic vesicles per terminal was significantly decreased when CBC preparations were incubated with either of the PLA_2_ toxins. In contrast, the number of synaptic vesicles relative to the area of the terminal was significantly reduced in preparations incubated with Bjme-TX-II but not with Bjme-TX-I. Synaptophysin expression is related to SV integrity [19] and its absence in preparations incubated with both toxins is in conformity with the reduction of vesicles in the terminal endings and the blockade of twitch tension in preparations treated with the toxins. On the other side, the reduction of the area of the nerve terminal seems to indicate that the presynaptic action of the two toxins is beyond the effect on the expression of the presynaptic proteins, the exact nature of which is obscure.

Snake presynaptic PLA_2_ neurotoxins (SPANs) paralyze the neuromuscular junctions (NMJs) in vertebrate skeletal muscles by reducing the content of SV. Studies using primary neuronal cultures show synaptic swelling, with surface exposure of the luminal domain of the synaptic vesicle protein synaptotagmin I, and exocytosis of neurotransmitters. Other studies using an equimolar mixture of lysophospholipids and fatty acids, which mimics the biological effects of SPANs, indicate a possible role of local lipid changes in SV release [19].

In this study, the 10 *μ*g/mL concentration of the toxins also induced alterations in the muscle fibres. It is likely that the onset of changes was triggered by damage of the phospholipid bilayer of the fibre membrane as inferred by histological and ultrastructural findings. Neuromuscular blockade and/or muscle damage, as induced by BmjeTX-I and BmjeTX-II in CBC preparations, was also reported in nerve-muscle preparations treated with other toxins, such as BthTX-I [8, 10] and BthTX-II [5] from* B*.* jararacussu* venom and BnpTX-I and BnpTX-II toxins [9] from* B. (neuwiedi) pauloensis* venom. Muscle damage inferred by rapid elevation of plasma CK activity in mice was also observed by Ponce-Soto et al. [17]* in vivo* with the two toxins here investigated. These results revealed that BmjeTX-I and BmjeTX-II PLA_2_s displayed a myotoxic effect, as the majority of venoms and toxins were isolated from* Bothrops* snakes, which is not associated with impairment of the nicotinic receptors of the postsynaptic sarcolemma.

Also, muscle ultrastructural alterations as those induced by BmjeTX-I and BmjeTX-II are similar to those described by Rodrigues-Simioni et al. [20] in frog nerve-muscle preparations incubated with* B*.* jararacussu* venom. Moreover, the changes in the intramuscular motor nerve axons are additional evidence of the neurotoxic action of the two PLA_2_s.

## 5. Conclusion

The absence of proteins involved in synaptic vesicles integrity (synaptophysin), vesicle docking, and transmitter exocytosis (synaptophysin, synaptobrevin, and SNAP25), in preparations incubated with BmjeTX-I and BmjeTX-II, is direct and strong evidence supporting our hypothesis that BmjeTX-I and BmjeTX-II affect neurotransmitter release machinery by preventing the docking of synaptic vesicles to the axolemma of the nerve terminal. The study reinforces the hypothesis that the lack of these proteins was responsible for the neurotoxicity caused by both toxins from* Bothrops marajoensis* venom.
